# Decreased Tissue Sodium Concentration in Suspected Prostate Cancer Detected by Internal-Reference ^23^Na MRI: A Prospective Exploratory Study

**DOI:** 10.3390/diagnostics16071064

**Published:** 2026-04-01

**Authors:** Anne Adlung, Niklas Westhoff, Daniel Hausmann, Stefan O. Schoenberg, Dominik Nörenberg, Frank G. Zöllner, Fabian Tollens

**Affiliations:** 1 Computer Assisted Clinical Medicine, Department of Radiation Oncology, Medical Faculty Mannheim, Heidelberg University, 68167 Mannheim, Germanyfrank.zoellner@medma.uni-heidelberg.de (F.G.Z.); 2 Mannheim Institute for Intelligent Systems in Medicine, Medical Faculty Mannheim, Heidelberg University, 68167 Mannheim, Germany; 3 Department of Radiology, New York University Langone Health, New York City, NY 10016, USA; 4 Department of Urology and Urosurgery, Mannheim University Medical Centre, 68167 Mannheim, Germany; niklas.westhoff@umm.de; 5 Department of Radiology, Klinik Hirslanden, 8032 Zurich, Switzerland; 6 Department of Radiology and Nuclear Medicine, Mannheim University Medical Centre, 68167 Mannheim, Germany; stefan.schoenberg@umm.de (S.O.S.); dominik.noerenberg@umm.de (D.N.); 7 Interdisciplinary Center for Scientific Computing, Heidelberg University, 69120 Heidelberg, Germany

**Keywords:** sodium MRI, ^23^Na MRI, prostate cancer, tissue sodium concentration, quantitative imaging

## Abstract

**Objectives**: To evaluate Sodium Magnetic Resonance (^23^Na MR) images of prostate cancer (PCa) and quantify tissue sodium concentration (TSC) based on internal references. **Methods**: Forty-six patients with clinically suspected prostate cancer were included into a prospective study. The patients underwent multiparametric MRI and an additional ^23^Na MRI examination of the prostate, performed at 3T (Magnetom Skyra, Siemens Healthineers, Erlangen, Germany) using a dual-tuned ^1^H/^23^Na body-coil (Rapid Biomedical, Rimpar, Germany) to acquire a 3D radial density-adapted ^23^Na sequence. Three-dimensional regions of interest (ROI) were defined within the femoral blood vessels, which were used as an internal reference for TSC quantification. Prostate zones and tumor ROIs were defined and TSC was evaluated for each ROI. **Results**: TSC quantification based on femoral blood vessels demonstrated good stability since mean absolute TSC difference between right and left regions of interest in the blood vessels was 3.3 ± 2.2 mM. TSC in the peripheral zone (40.7 ± 6.0 mM) was significantly higher than in the transition zone (37.5 ± 5.7 mM). Nine suspicious lesions (PI-RADS 4 and 5) were identified in eight men, all with biopsy-proven PCa with Gleason scores of ≥3 + 3. TSC in prostate cancer was significantly lower than in contralateral healthy regions, 32.2 ± 5.5 mM and 36.1 ± 3.9 mM, respectively (*p* = 0.018). **Conclusions**: TSC quantification of prostate tissue based on internal references is feasible and reliable. TSC was significantly decreased within prostate cancer, which represents a quantitative imaging biomarker that could potentially improve PCa characterization and risk stratification.

## 1. Introduction

Prostate cancer (PCa) is the most common non-cutaneous malignant tumor in men, and it is the second most cancer-related cause of death in men worldwide [1]. The potential of multiparametric MRI (mpMRI) of the prostate within clinical workflows is the detection and staging of PCa to enable image-guided biopsies and to increase the rate of positive biopsies [2]. Concerns on overdiagnosis and overtreatment of indolent PCa have directed attention to improving non-invasive, imaging-based characterization of indeterminate lesions and cancer mimics that could undergo unnecessary diagnostic work-up [3].

Various imaging techniques are emerging that hold promise in improving prostate tissue characterization, such as luminal water imaging, hybrid multidimensional MRI, restriction spectrum imaging and MR fingerprinting [4,5,6,7]. Beyond MRI, several complementary modalities are under active clinical investigation for prostate cancer detection and characterization, including shear wave elastography (SWE), which exploits differences in tissue stiffness to identify malignant lesions [8]; contrast-enhanced ultrasound (CEUS), which visualizes tumor-associated neovascularization to improve targeted biopsy yield [9]; and prostate-specific membrane antigen PET/CT (PSMA-PET/CT), which offers whole-body molecular staging with superior sensitivity for locoregional and metastatic disease compared to conventional imaging [10].

Sodium Magnetic Resonance Imaging (^23^Na MRI) exploits the nuclear spin-3/2 property of the sodium nucleus, which—unlike protons—exhibits a quadrupolar moment that interacts with local electric field gradients in biological tissue, resulting in biexponential T2* relaxation with a fast component (T2f ≈ 0.5–5 ms) and a slow component (T2s ≈ 10–65 ms) [11]. The gyromagnetic ratio of ^23^Na (γ = 11.26 MHz/T) is approximately four times lower than that of ^1^H, and the in vivo sodium concentration in soft tissue (15–150 mM) is several orders of magnitude below that of tissue water protons, collectively resulting in an intrinsic signal-to-noise ratio (SNR) that is approximately 3000–20,000 times lower than that of conventional ^1^H MRI [12]. To compensate for this inherent SNR deficit, ^23^Na MRI requires dedicated multinuclear radiofrequency coils, ultra-short echo-time acquisition sequences, higher magnetic field strengths (≥3 T), and acquisition times of typically 10–20 min [13]. Despite these technical demands, ^23^Na MRI provides quantitative information on tissue sodium concentration (TSC), which reflects the balance between intracellular and extracellular sodium compartments, the extracellular volume fraction, and cellular density—parameters that are directly linked to cellular homeostasis and are altered in pathological states including malignancy [14].

Preliminary studies on ^23^Na MRI of the human prostate have demonstrated significantly altered sodium levels in PCa [15,16,17,18,19,20]. Therefore, ^23^Na MRI could improve detection of clinically significant PCa in distinction from benign entities and may serve as an additional quantitative imaging biomarker.

For abdominal ^23^Na MRI, precise TSC quantification requires references with a known sodium concentration. External reference phantoms placed within the field of view (FoV) are well-established and considered state-of-the-art, similarly to other entities such as neuroimaging [21,22]. However, quantification accuracy is impaired by the distance between reference and investigated tissue as it is affected by field and coil inhomogeneity. Quantifying the TSC based on internal references that are in spatial proximity to the prostate represents an alternative approach and could make results more stable and less prone to coil and field-induced inaccuracies.

The femoral blood vessels have an adequate diameter to be easily distinguishable on prostate MR images, and TSC in the human blood has been quantified previously [23,24,25]. Thus, femoral blood vessels could serve as reference for TSC quantification on ^23^Na MR images of the prostate.

We hypothesized that using internal references to quantify TSC of the prostate is feasible may help to characterize prostate lesions and could prospectively improve risk-stratification.

## 2. Materials and Methods

**Figure 1:**
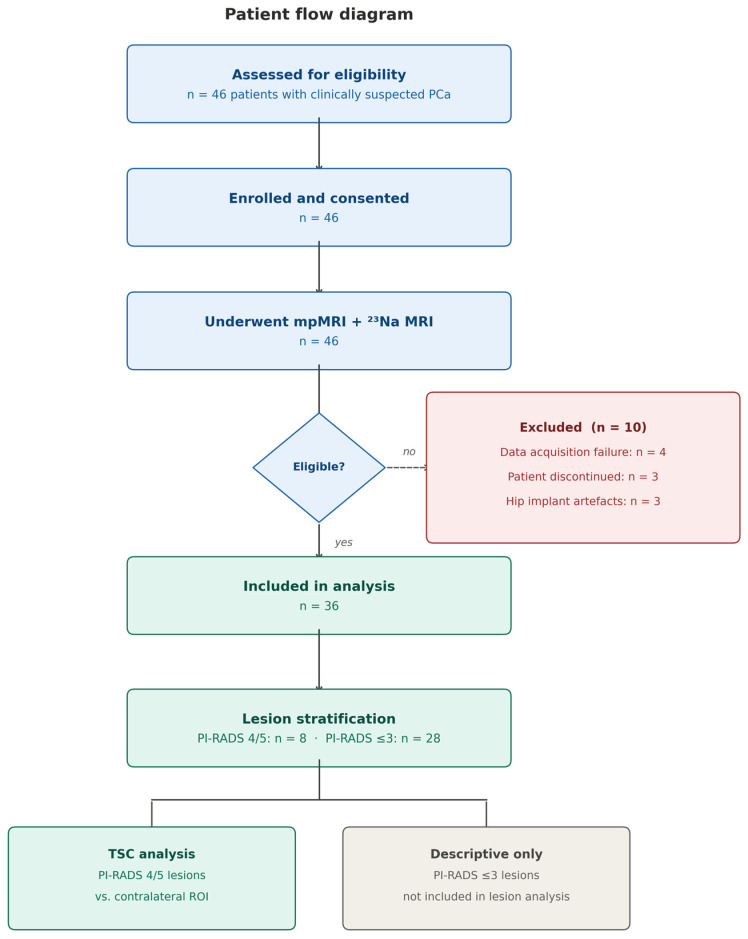
Patient flow diagram. Of 46 patients enrolled, 10 were excluded prior to analysis (4 due to data acquisition failure, 3 due to premature termination of the examination, and 3 due to severe image artifacts caused by hip implants). The remaining 36 patients were included in the final analysis. Patients with PI-RADS 4 or 5 lesions (*n* = 8) with biopsy-confirmed PCa were included in the primary lesion-level TSC analysis; patients with PI-RADS ≤ 3 were not included in the lesion analysis.

The local ethics committee granted approval for this prospective study. A total of 46 patients with clinically suspected PCa were enrolled between March 2020 and October 2021, provided written informed consent, and underwent standard mpMRI as well as an additional ^23^Na MRI examination of the prostate. Exclusion criteria were previous therapeutic interventions for PCa and contraindications to MRI. Of the 46 enrolled patients, 10 were subsequently excluded prior to analysis: four due to data acquisition failure, three due to premature termination of examination, and three due to severe image artifacts caused by hip implants. The remaining 36 patients were included in the final analysis. The patient flow is summarized in Figure 1.

### 2.1. Data Acquisition

Prior to the study protocol, each patient underwent a standard mpMRI examination of the prostate according to the PI-RADS v2.1 guidelines [26]. Gadolinium-based contrast agent (Dotarem, 0.1 mL/kg body weight) was administered as part of the mpMRI protocol; based on previous findings, this did not impact subsequent TSC quantification [27]. The study protocol was acquired at 3 T (Magnetom Skyra, Siemens Healthineers, Erlangen, Germany) using a dual-tuned ^1^H/^23^Na body-coil (Rapid Biomedical, Rimpar, Germany). This coil consisted of a birdcage-like ^23^Na transmit coil with a fixed frame, 16 ^23^Na receive channels and six ^1^H receive channels located on an array flex surface coil, and used the scanner’s built-in ^1^H transmit channels.

The study protocol comprised two sequences: a 3D radial density-adapted ^23^Na sequence [28] and a ^1^H T2-weighted turbo-spin-echo (T2w TSE). The ^23^Na sequence was acquired with TR/TE = 120/1.2 ms, a flip angle of 90°, 8000 spokes with 384 samples each, a gradient amplitude of 3.6 mT/m, a slewrate of 170 T/m·s and a bandwidth of 50 Hz/Pixel, yielding a nominal isotropic resolution of 5.1 × 5.1 × 5.1 mm^3^ and a total acquisition time of 16 min. The ^1^H T2w TSE was acquired with TR/TE = 4710/108 ms, a flip angle of 160°, a FOV of 250 × 250 mm^2^, an in-plane resolution of 0.7 × 0.7 mm^2^, 36 slices with a slice thickness of 3.0 mm, and an acquisition time of 3 min and 57 s. The T2w TSE served as the anatomical reference for all subsequent segmentation and co-registration steps.

Two radiologists with 10 and 3 years of experience in prostate imaging performed all image readings and segmentations in a consensus session using MITK (German Cancer Research Center, Heidelberg, Germany). PI-RADS v2.1 scores were assigned for each patient, and the whole prostate (WP) and peripheral zone (PZ) were manually segmented on the T2w SE images. The transition zone (TZ) mask was derived by subtracting the PZ mask from the WP mask. Focal suspicious lesions (PI-RADS 4 and 5) were individually segmented, and contralaterally mirrored ROIs in healthy prostate tissue were defined for each lesion to serve as paired reference regions. PI-RADS 3 lesions were not included in the lesion-level analysis, as prostatectomy specimens were unavailable for imaging-pathology correlation and their malignant potential could therefore not be reliably determined (see Section 4.1 for discussion). Five of the eight patients with suspicious lesions received targeted biopsies, and three received systematic biopsies.

### 2.2. Image Reconstruction

**Figure 2:**
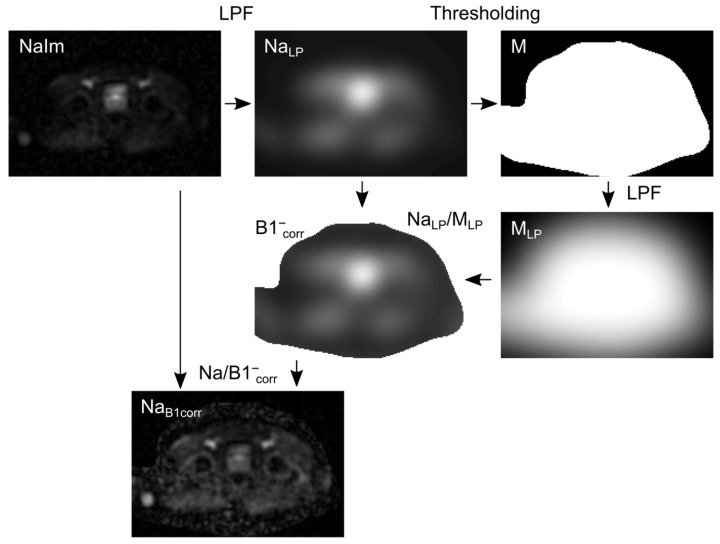
Schematic illustration of the B1^-^ correction of the ^23^Na MR image using a low-pass filter. The filter is applied to the acquired ^23^Na MR image NaIm, which was reconstructed using the adaptive coil combination and generated Na_LP_. The mask M was obtained via thresholding of Na_LP_. Another low-pass filter was applied to M, which generated M_LP_. The correction map B1^-^_corr_ was calculated by the ratio of Na_LP_ to M_LP_. Na_B1corr_ is the B1^-^corrected image and was calculated by the division of NaIm by B1^-^_corr_. The algorithm was adapted from Lachner et al. [23].

All ^23^Na MR image reconstruction was performed in MATLAB 2018a and followed a sequential pipeline comprising per-channel reconstruction, adaptive coil combination, and B1^-^ inhomogeneity correction, as schematically illustrated in Figure 2.

In the first step, each of the 16 ^23^Na receive channels was reconstructed individually. Reconstruction applied a Hanning filter in k-space and a Kaiser–Bessel window with a width of 4. A zero-filling factor of 2 was additionally applied, increasing the apparent isotropic resolution to 2.55 × 2.55 × 2.55 mm^3^.

In the second step, the 16 individual channel images were combined using adaptive coil combination (ACC), which weights each channel proportionally to its acquired signal intensity, thereby optimizing the composite image for the signal-to-noise ratio [29].

In the third step, the ACC-combined image was corrected for B1^-^ receive field inhomogeneities using a low-pass filter (LPF)-based approach adapted from Lachner et al. [30]. Briefly, a 3D cubic Gaussian filter (standard deviation $\;\sigma_{1}$ = 10 voxels, ~25 mm; filter size 41 voxels) was applied to the ^23^Na image (Na_Im_), yielding a smoothed image Na_LP_. A binary mask M was obtained by thresholding Na_LP_, and a second 3D cubic Gaussian filter ($\;\sigma_{2}$ = 20 voxels, ~50 mm; filter size 81 voxels) was applied to M, yielding M_LP_. The B1^-^ correction map B1^-^_corr_ was computed by element-wise division of Na_ImLP_ by M_LP_, and the corrected image ${Na}_{B1corr}$ was obtained accordingly. The complete B1^-^ correction pipeline is illustrated schematically in Figure 2.

### 2.3. TSC Quantification

Following image reconstruction, the ^23^Na MR image was co-registered to the T2w TSE in MATLAB 2018a. Co-registration was achieved by automatic slice positioning alignment and re-sampling of the ^23^Na MR image to the TSE resolution, supplemented by a small anterior manual shift to achieve optimal anatomical alignment. After co-registration, all segmentation masks defined on the T2w TSE—including WP, PZ, TZ, focal lesions and contralateral ROIs—were directly transferable to the ^23^Na space.

**Figure 3:**
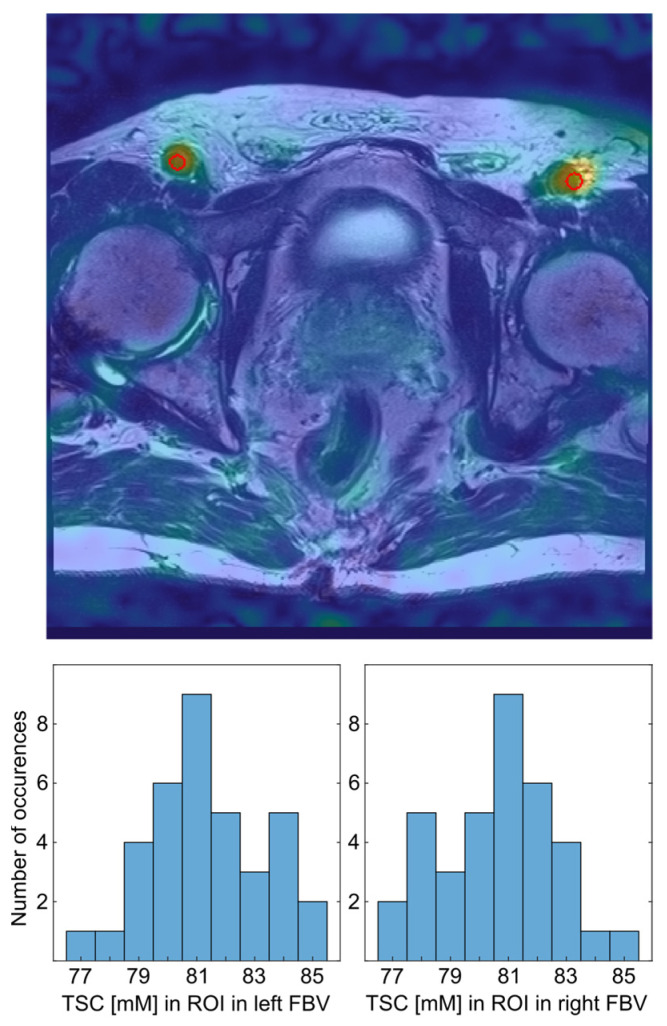
Transverse slice of the co-registered ^23^Na MR image of one patient with the segmentation of a ROI (red circle) in the left and right the femoral blood vessels (FBV), which were used for the quantification. The ^23^Na MR image is shown as an overlay over the T2w TSE and the segmented regions are encircled in red (**top**). Histogram of the TSC levels in the left and right FBV, respectively (**bottom**).

**Figure 4:**
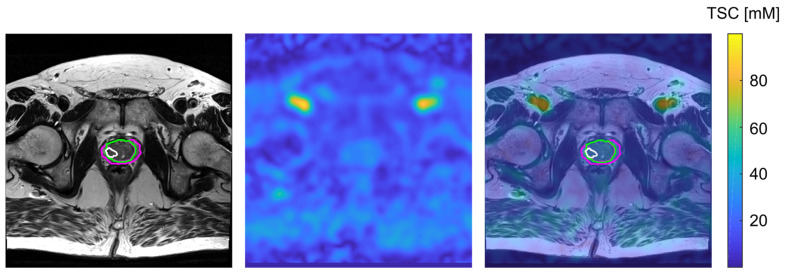
Transverse slice of the T2w TSE MR image of one patient with the segmentation of PZ (pink), TZ (green), and one lesion (white) within the TZ (**left**), the corresponding co-registered and quantified ^23^Na MR image (**middle**) and the overlay of both images, including the segmented regions (**right**).

For TSC quantification, internal reference regions were defined within the femoral blood vessels (FBV), which exhibit high ^23^Na signal intensity relative to prostate tissue and are spatially proximate to the gland. Three-dimensional ROIs were placed bilaterally within the right and left iliac-femoral blood vessel transition on the ^23^Na image, guided by anatomical landmarks visible on the co-registered T2w TSE (Figure 3). The use of co-registered T2w TSE images for ROI placement is illustrated in Figure 3, and representative co-registered ^23^Na and T2w images with transferred segmentations masks are shown in Figure 4.

TSC was quantified using a linear fit of the mean signal intensity (SI) within each ROI. Based on published ^23^Na MRI measurements reporting blood TSC in the range of 71–85 mM [23,24,25], and consistent with the most recent value reported by Lott et al. (81 ± 7 mM [25]), a fixed blood TSC of 81 mM was assumed for the FBV reference. SI within the FBV ROIs was corrected for T1 and monoexponential T2* relaxation effects using T1(blood) = 31.9 ms and T2*(blood) = 20.1 ms, as reported by Konstandin et al. [24]. For prostate tissue, relaxation time corrections were applied using T1(prostate) = 38.8 ms, T2f(prostate) = 6.8 ms and T2_s_(prostate) = 14.8 ms, accounting for the biexponential T2* decay characteristic of biological tissue sodium.

Mean TSC was calculated within WP, PZ, TZ, individual focal lesions, and their contralateral ROIs. To minimize partial volume effects (PVE) arising from the limited spatial resolution of the 23Na acquisition, the outer borders of all segmentation masks were eroded prior to TSC calculation. An erosion distance of 5.2 mm—corresponding to the nominal voxel size—was applied for WP and TZ. For the PZ, which was too small to accommodate full erosion, a reduced cut-off of 2.6 mm was used. No erosion was applied to focal lesions and their contralateral ROIs, given their small size; the potential impact of residual PVE on these regions is discussed in Section 4.1. The stability and reproducibility of the internal reference method were evaluated by comparing mean TSC, within-ROI standard deviation, and left–right differences across the bilateral FBV ROIs.

### 2.4. Statistical Analysis

Statistical analyses were performed in Python 3.10 (scipy.stats library, version 1.11). Prior to inferential testing, the one-sample Kolmogorov–Smirnov test confirmed that absolute TSC values followed a normal distribution across all evaluated tissue regions, justifying the use of parametric tests.

A two-tailed paired Student’s *t*-test was applied to compare TSC between PZ and TZ across all 36 patients and to compare TSC between confirmed PCa lesions and their contralateral healthy ROIs. The same test was used to assess bilateral symmetry of TSC within the FBV reference ROIs. The Pearson correlation coefficient was calculated to assess the relationship between TSC and ADC values within WP, PZ, and TZ, and within the confirmed PCa lesions. A *p*-value of <0.05 was considered statistically significant for all tests. Results are reported as mean ± standard deviation (SD) unless otherwise stated.

A post hoc power analysis was performed for the primary lesion-level comparison (PCa lesions versus contralateral ROIs) using the non-central t-distribution for a two-tailed paired *t*-test (α = 0.05). Cohen’s d was back-calculated from the observed t-statistic and sample size; the corresponding retrospective power and the minimum sample size required to achieve 80% and 90% power at the observed effect size are reported in Section 4.1.

## 3. Results

A total of 46 male patients with a clinically suspected PCa was included in the study. The data of 36 patients were analyzed; the remaining ten patients were excluded due to failure of data acquisition (*n* = 4), the patient not feeling capable of completion of the measurement (*n* = 3), or because the patient had a hip implant and the resulting image artifacts were too severe for reliable image analysis (*n* = 3). Mean age of the included patients was 66.5 ± 7.5 years with mean PSA levels of 9.1 ± 6.1 ng/mL. Of those, five were rated with a PI-RADS 5 lesion, three with a PI-RADS 4 lesion, and the remaining 28 patients were rated with lower-ranking lesions or did not show any suspicious lesion visible on mpMRI (Table 1). All patients with suspicious lesions (PI-RADS 4 or 5 lesions) had biopsy-proven PCa with Gleason scores of ≥3 + 3. Six patients with lower ranking PI-RADS scores also had a biopsy positive for PCa; the remaining patients were lost to follow-up.

All PI-RADS 5 lesions (five patients with six lesions) as well as one PI-RADS 4 lesion were located within the PZ, with one patient presenting with multifocal PCa with two segmentable focal lesions (both Gleason score 3 + 4). The two remaining patients with a PI-RADS 4 lesion (Gleason scores 3 + 3 and 3 + 4, respectively) had their lesions in the TZ. Absolute TSC quantification was performed for all patients and within all ROIs (Table 2).

**Figure 5:**
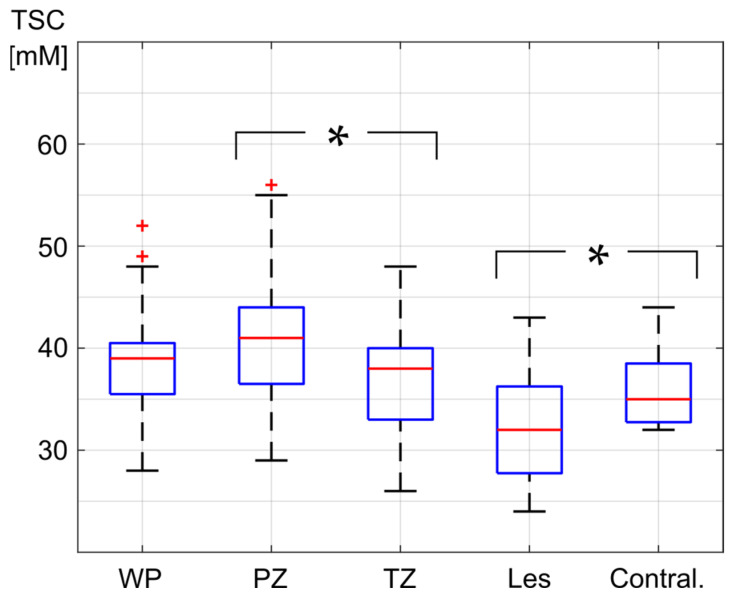
Mean absolute tissue sodium concentration (TSC) in the whole prostate (WP), peripheral zone (PZ), transition zone (TZ), and the segmented lesions (Les) as well as their contralateral regions (Contral.). The red line in the box depicts the median value, and the blue box’s top and bottom edges represent the 25th and 75th percentiles of the data, respectively. The whiskers extend to the most extreme data points. Statistically significant differences are indicated with a *.

Within the WP, all evaluated patients showed a mean absolute TSC between 28.5 and 52.2 mM, with a mean of 39.0 ± 5.3 mM. In the PZ, all patients showed a mean absolute TSC between 28.6 and 55.9 mM with a mean of 40.7 ± 6.0 mM, and in the TZ, all patients showed a mean absolute TSC between 25.8 and 48.4 mM with a mean of 37.5 ± 5.7 mM. The mean absolute TSC of all patients was significantly higher in the PZ than in the TZ (*p* = 0.0004), by a mean of 4.7 ± 3.5 mM (Figure 5).

Evaluation of the ROIs within the FBV—which served as TSC quantification references and were set to a mean absolute TSC of 81 mM—showed a mean absolute TSC of 80.4 ± 1.9 mM on the left side and a mean absolute TSC of 81.6 ± 1.9 mM on the right side. Mean absolute TSC difference between both sides was 3.3 ± 2.2 mM. There was no significant difference between absolute TSC within the FBV on the left side compared to the mean absolute TSC within the FBV on the right side (*p* = 0.076). Mean SD within the left FBV was 3.2 ± 1.3 mM, and mean SD in the right FBV was 3.9 ± 1.7 mM. Considering both FBV, mean SD of TSC within the entire ROIs was at 4.2 ± 1.3 mM.

For the seven segmented lesions in the PZ (from *n* = 6 patients), the mean absolute TSC levels ranged from 24.4 to 43.4 mM, with a mean of 32.1 ± 5.8 mM. For all patients, mean absolute TSC within the lesion(s) was lower than (*n* = 6) or equal to (*n* = 1) mean absolute TSC within the patient’s PZ. Furthermore, mean TSC of the PCa lesions was lower than of the contralateral ROI for five out of seven lesions whereas it was equal for two of the lesions.

The two lesions in the TZ showed mean TSC levels of 36.9 ± 3.6 mM and 28.4 ± 1.8 mM, which were lower than mean TSC in the healthy TZ for both patients (mean TSC with TZ was 40.2 ± 4.9 and 37.7 ± 3.8 mM, respectively). The same was applicable when comparing the TSC in the lesions to their contralateral ROIs.

**Figure 6:**
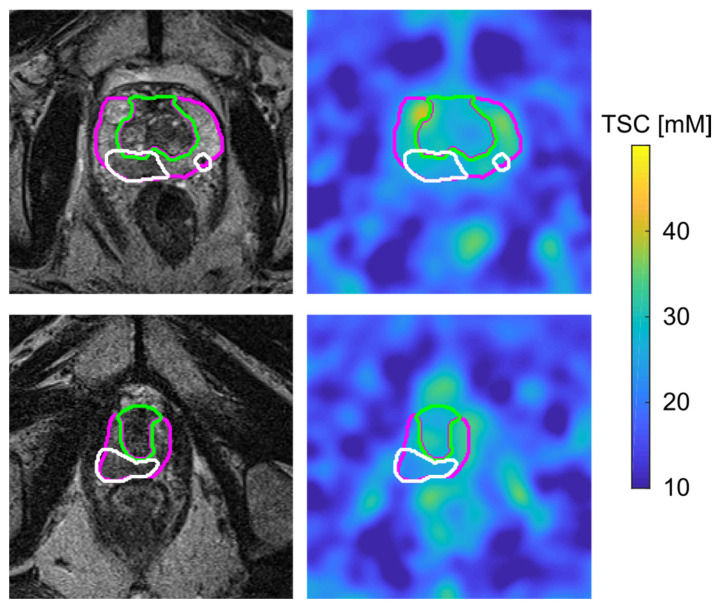
Transverse slice of MR images of two patients with one (**bottom**) and two (**top**) lesions in the peripheral zone of the prostate. The T2w TSE images are shown on the left, including the segmentation of the peripheral zone (pink), transition zone (green) and lesions (white), and the co-registered and quantified ^23^Na MR image are shown on the right, including the transferred segmentation masks of the regions of interest.

Overall, TSC in the PCa lesions was significantly lower than TSC in the contralateral healthy ROIs (*p* = 0.0181). The PZ, TZ and lesion segmentations are depicted on the T2w and on the quantified ^23^Na MR images of two patients in Figure 6. There was a positive correlation between TSC and ADC values within the evaluated PCa lesions, which was, however, not statistically significant (R = 0.67, *p* = 0.0690) within our small patient collective.

## 4. Discussion

Our prospective study in newly diagnosed men with suspected PCa confirmed utilization of internal references to be feasible for quantifying TSC of prostate tissue and suspicious prostate lesions. Significantly reduced TSC was found within PCa lesions, representing a potential quantitative biomarker for improved characterization of PCa.

Internal references represent an alternative approach in TSC quantification in the abdomen, which is commonly performed based on reference phantoms placed within the FoV. This may cause severe inaccuracies within the patient’s abdomen [31,32] as the used multi-channel abdominal coil has no homogeneous B1^—^field, and the principle of reciprocity does not apply [33], which warrants for further corrections [34,35,36]. Inaccuracies were reduced by using ACC, which decreases the influence of channels with weaker signals. A LPF was used for B1^-^ corrections, smoothing the image signal [30].

Although the assumption of a relatively homogeneous B1^+^-field was appropriate because of the birdcage structure of the circular transmit coil, inhomogeneities might remain, particularly in the peripheral FoV. Thus, TSC quantification of the prostate using external reference phantoms with a larger distance to the investigated tissue are afflicted with considerable drawbacks and were not considered appropriate. The femoral blood vessels as quantification references are less prone to inaccuracies caused by B1 inhomogeneities because of their spatial proximity to the investigated tissue.

Considering the TSC of the right and left FBV, variation in absolute TSC was low and absolute differences between both sides were not significant, indicating sufficient stability of the quantification method. ADC values represent the tissue’s diffusion and, consequently, decrease with an increasing cell density. The same is applicable for TSC, which also decreases with increasing cell density because of the lower intracellular sodium concentration, compared to extracellular values. Correlation between TSC and ADC values in the cancer lesions was positive, although not significant. If the existing trend could be observed for a lager cohort, this could further support the reliability of the results.

There are only a limited number of studies on ^23^Na MRI in the human prostate, particularly in PCa. In studies investigating the healthy prostate tissue, Hausmann et al. reported TSC values ranging from 24 to 70 mM in the PZ and from 34 to 85 mM in the TZ [19]. In contrast, Farag et al. reported higher values, with TSC ranging from 51 to 92 mM in the PZ and from 72 to 98 mM in TZ [18].

More recent studies have focused on TSC in PCa. Investigations by Barrett et al. reported TSC levels of approximately 33 and 34 mM in TZ and 39 mM in PZ and additionally observed elevated TSC within the PCa lesions, with mean values of 42 and 43.1 mM [15,17]. In 2019, Broeke et al. also found increased TSC values in PCa compared to healthy prostate tissue, particularly in lesions with higher Gleason scores [16].

Most recently, Tan et al. reported mean TSC levels of 78 mM of noncancerous PZ tissue and 81 mM of noncancerous TZ tissue in PCa patients [20]. TSC levels were significantly lower in cancers compared to noncancerous tissue only in the TZ but not in the PZ.

The zonal distribution of PCa lesions observed in our cohort is consistent with established epidemiological data, with the majority of lesions arising in the PZ, which accounts for approximately 70% of all PCa cases [37,38]. Importantly, our study demonstrated significantly higher baseline TSC in the healthy PZ compared to the TZ (40.7 ± 6.0 mM vs. 37.5 ± 5.7 mM, *p* = 0.0004), a finding that has direct implications for the definition of diagnostic thresholds in TSC-based lesion characterization. Since TSC in PCa lesions is evaluated relative to the surrounding healthy tissue of the respective zone, zone-specific reference values and corresponding TSC thresholds will likely be required for reliable lesion detection in both compartments. This notion is further supported by Tan et al., who recently reported that TSC differences between cancerous and noncancerous tissue reached statistical significance in the TZ but not in the PZ when analyzed as absolute values, highlighting the diagnostic challenge posed by the lower baseline TSC contrast in PZ cancer [20]. Future studies with adequate statistical power should therefore evaluate TSC thresholds separately for PZ and TZ lesions and assess whether zone-specific TSC analysis can improve sensitivity and specificity for clinically significant PCa detection, complementing the established zone-specific diagnostic criteria of PI-RADS v2.1.

The presented study showed mean absolute TSC values of all patients to be 40 ± 5 mM in the WP. TSC levels within the PZ were significantly higher than TSC levels in the TZ. The quantitative results are within the broad range of previously reported values. They are similar to Barrett et al.’s findings and at the lower end of findings from Hausmann et al. but they are substantially lower than the values that Farag et al. and Tan et al. have reported [15,17,18,19,20]. Furthermore, the inter-subject SD within all evaluated tissues was relatively low, indicating stability within the quantification method.

In our study, TSC in the PCa lesions was markedly lower than TSC in healthy prostate tissue. The discrepancy between the elevated TSC in PCa reported by Barrett et al. and Broeke et al. on the one hand, and the decreased TSC observed in the present study and by Tan et al. on the other, warrants careful methodological consideration and cannot be attributed to a single variable. Barrett et al. employed a dedicated dual-tuned ^1^H/^23^Na endorectal receive coil in conjunction with a clamshell transmit coil at 3 T, with phantom vials of known sodium concentration integrated directly into the endorectal coil assembly to enable absolute TSC quantification, and applied full T1 and biexponential T2* relaxation time corrections including inversion recovery sequences to separate intracellular sodium contributions [15,17]. Broeke et al. used an external broadband surface coil at 3 T and reported TSC as relative percent changes from healthy tissue (ΔTSC) rather than absolute values, subsequently co-registering all imaging data to Gleason-graded whole-mount prostatectomy histology—the only study in this field to demonstrate a statistically significant correlation between ΔTSC and Gleason score [16]. In contrast, the present study employed a 16-channel external birdcage transmit and flex array receive coil, quantified TSC using femoral blood vessels as internal references rather than external phantoms, and used systematic biopsy rather than prostatectomy as the histological reference standard. Tan et al. similarly used a completely external custom-built two-loop butterfly coil at 3 T and reported significantly lower TSC in PCa lesions compared to noncancerous tissue (ΔTSC − 14.1 ± 18.2%, *p* = 0.0002), with absolute TSC values substantially higher than those of the present study, likely reflecting differences in sensitivity correction methodology [20].

Three interrelated factors most plausibly explain the divergent TSC direction across studies. First, endorectal coils provide a highly inhomogeneous B1 receive field that peaks in the peripheral zone, where the coil is in closest proximity; phantom-based calibration vials integrated into the coil assembly share this proximity and thus may systematically overestimate TSC in peripheral tissue relative to the coil—potentially amplifying signals from high-vascular, high-extracellular-volume tumor regions and producing apparent TSC elevation. External coils, by contrast, provide a more homogeneous sensitivity profile across the whole gland, reducing this spatial bias. Second, the use of relative metrics (ΔTSC) by Broeke et al. and Tan et al. circumvents absolute calibration errors but precludes direct comparison of numerical TSC values across studies. Third, and most biologically relevant, the histological reference standard is critical: prostatectomy-based co-registration with whole-mount specimens, as used by Barrett et al. and Broeke et al., enables precise spatial mapping of tumor infiltration patterns including the extracellular matrix and vascularity of the tumor microenvironment, whereas systematic and targeted biopsies—as used in the present study—introduce spatial uncertainty and may preferentially sample regions of high cell density and reduced extracellular volume, thereby biasing towards lower TSC. A structured methodological comparison of all published ^23^Na MRI studies in the human prostate is provided in Supplementary Table S1. Taken together, these methodological differences are likely sufficient to explain the opposing TSC findings and underscore the urgent need for standardized acquisition, calibration, and histological validation protocols before ^23^Na MRI can be evaluated across centers.

Previously, TSC was reported to be elevated within various malignant tumors throughout the body [14,39,40]. It is hypothesized that an increased cell metabolism in tumor cells increases the intracellular sodium concentration and that an increased vascularization of tumors increases their relative extracellular volume. However, tumors also present with a higher cell density which comes along with a decrease in extracellular volume fraction causing reduced ADC values. The same could apply for TSC levels, which also decrease with an increasing cell density due to the lower intracellular compared to extracellular sodium concentration. This might offer an explanation for the observed decreased TSC levels in cancer lesions as well as for the positive, yet not significant, correlation between ADC and TSC values in the cancer lesions.

### 4.1. Clinical Perspectives

From a clinical perspective, the integration of ^23^Na MRI into routine prostate cancer imaging workflows appears challenging. A full mpMRI examination according to PI-RADS v2.1 requirements typically requires approximately 15–30 min of acquisition time, depending on scanner hardware and prostate size [41]. The ^23^Na MRI sequence used in the present study added approximately 16 min of acquisition time, representing a significant increase in total examination duration that is comparable to the addition of other emerging functional sequences currently under investigation. Dedicated dual-tuned ^1^H/^23^Na radiofrequency coils, as employed in this study, are a prerequisite and currently limit broad multi-center availability; however, ongoing hardware developments may reduce this barrier in the future [11].

Beyond lesion detection, quantitative TSC may hold particular promise as a non-invasive biomarker in the context of active surveillance (AS). Gleason score upgrading—the reclassification of a biopsy-confirmed low-grade tumor to a higher-grade tumor—remains one of the challenges in selecting men for AS, with upgrading rates of up to 44.6% reported in Gleason score 3 + 4 cohorts after prostatectomy [42]. Given that TSC reflects cell density, extracellular volume fraction, and cellular metabolic activity—all of which are expected to change with tumor dedifferentiation—quantitative TSC maps could prospectively complement mpMRI in identifying lesions at risk of upgrading, thereby informing the intensity and frequency of surveillance biopsy schedules.

### 4.2. Limitations

The present study has several limitations that must be considered when interpreting the findings. First, and most importantly, the limited cohort size—36 analyzable patients, of whom only eight had PI-RADS 4 or 5 lesions with biopsy-confirmed PCa—precludes definitive conclusions regarding diagnostic thresholds, sensitivity, or specificity of TSC-based lesion characterization. A post hoc power analysis for the primary comparison (TSC in confirmed PCa lesions versus contralateral healthy tissue, two-tailed paired *t*-test, α = 0.05) revealed an observed effect size of Cohen’s d = 0.99, corresponding to a retrospective power of 73.7% for the nine lesions included in this analysis. Although this exceeds the commonly cited threshold of 70% and is consistent with the achieved statistical significance (*p* = 0.018), this study remained underpowered relative to the conventional 80% threshold; attaining 80% power with the observed effect size would require at least 11 lesions, and 90% power would require approximately 15 lesions. For a medium effect size (d = 0.50), which may be more representative of subtle TSC differences in clinically equivocal lesions, 34 lesions would be needed to achieve 80% power. The secondary comparison between PZ and TZ across all 36 patients was adequately powered (Cohen’s d = 1.34, retrospective power > 99%), consistent with the highly significant difference observed (*p* = 0.0004). These results confirm the exploratory nature of the primary lesion-level analysis and underscore the need for substantially larger prospective cohorts to draw robust conclusions regarding the diagnostic utility of TSC in individual lesion characterization. This is inherent to the early-stage, prospective feasibility design of the study; nevertheless, prospective studies with substantially larger cohorts, mandatory stratification by Gleason grades, and prostatectomy-based imaging-pathology correlation using whole-mount specimens are required to validate the observed TSC differences and to establish clinically meaningful TSC thresholds before this technique can be considered for integration into structured clinical decision pathways.

Second, a further methodological constraint concerns the exclusion of PI-RADS 3 lesions from the lesion-level TSC analysis. PI-RADS 3 lesions are by definition equivocal, and their management remains one of the most contested topics in prostate MRI. PCa is detected in about 31% of PI-RADS 3 lesions, while only 14% of PI-RADS 3 lesions are clinically significant [43]. Without prostatectomy-based imaging-pathology co-registration—which was not available in the present study—the histological ground truth of individual PI-RADS 3 lesions cannot be established with sufficient confidence. Classifying these lesions as either cancerous or benign based on systematic biopsy alone would have introduced unacceptable diagnostic uncertainty into the TSC analysis, as TSC differences between truly malignant and truly benign PI-RADS 3 lesions could not be reliably determined. The decision to restrict the primary lesion-level analysis to PI-RADS 4 and 5 lesions with biopsy-confirmed PCa was therefore methodologically justified as a conservative approach to ensure the integrity of the comparisons reported.

We acknowledge, however, that this exclusion constitutes a source of selection bias with direct implications for the real-world diagnostic applicability of ^23^Na MRI. In clinical practice, the precise characterization of PI-RADS 3 lesion and identification of the subset harboring clinically significant cancer represents an area of great diagnostic need. If ^23^Na MRI-derived TSC values were ultimately shown to stratify malignant from benign PI-RADS 3 lesions, the clinical impact would be considerably greater than for PI-RADS 4/5 lesions, which already carry a high pre-test probability of clinically significant disease. Future studies should therefore be specifically designed to include PI-RADS 3 lesions with mandatory prostatectomy-based co-registration as the reference standard, enabling a rigorous evaluation of whether TSC-based characterization can meaningfully reduce unnecessary biopsies in this diagnostically challenging population.

Third, the use of femoral blood vessels as internal quantification references, while reducing susceptibility to B1 field inhomogeneities compared to external phantom-based approaches, introduces patient-specific confounders into TSC quantification. The assumed blood TSC of 81 mM [18] is subject to variability driven by individual differences in serum sodium concentration (typically 135–145 mM) and haematocrit (40–50% in healthy men) [44,45]. Propagated through the linear TSC calibration to prostate tissue (mean TSC ~40 mM), this blood TSC variability introduces a systematic uncertainty of approximately ±2 mM (1σ) or ±4 mM (95%) in absolute prostate TSC values—corresponding to approximately 5% of the mean whole-prostate TSC reported in this study. A dedicated validation study in healthy controls including individual measurement of serum sodium and haematocrit, systematically investigating the reliability and reproducibility of this internal-reference quantification approach, would therefore be of considerable interest.

Fourth, quantitative ^23^Na MRI is inherently susceptible to partial volume effects (PVE), arising from the relatively low nominal spatial resolution of the acquisition (5.1 × 5.1 × 5.1 mm^3^). Although PVEs were mitigated by excluding the outer borders of each segmentation mask and restricting analysis to the central tissue volume, the required cut-off distance had to be adapted according to the size of the investigated region. As a consequence, smaller ROIs—in particular, the focal suspicious lesions and their contralateral counterparts—were likely more strongly affected by residual PVEs than larger regions such as the whole prostate or the transition zone. This size-dependent PVE susceptibility may have introduced a systematic underestimation of TSC differences between lesions and surrounding tissue and should be addressed in future studies through higher-resolution acquisitions or dedicated PVE correction algorithms.

Finally, the image reconstruction and B1^-^ correction pipeline, while described with sufficient parametric detail to allow replication, was implemented in MATLAB and requires a dedicated dual-tuned ^1^H/^23^Na radiofrequency coil, currently limiting broad multi-center applicability. Standardization of acquisition and post-processing protocols across sites will be a prerequisite for the multi-center validation studies necessary to establish ^23^Na MRI as a clinically translatable imaging biomarker in prostate cancer.

## 5. Conclusions

In summary, this prospective feasibility study demonstrates that internal referencing using femoral blood vessels is a viable approach for absolute TSC quantification in the human prostate at 3 T, yielding stable and reproducible reference values with low inter-side variability. Absolute TSC values in the whole prostate, peripheral zone, and transition zone were consistent with the lower range of previously reported values, and TSC in the peripheral zone was significantly higher than in the transition zone across all patients, a finding relevant to the definition of zone-specific diagnostic thresholds in future studies.

Within the small cohort of biopsy-confirmed PCa lesions examined, TSC was decreased compared to contralateral healthy prostate tissue, suggesting that reduced sodium concentration—likely reflecting increased cell density and a corresponding reduction in extracellular volume fraction—may represent a quantitative imaging feature of prostate cancer detectable by ^23^Na MRI. A positive, albeit non-significant, correlation between TSC and ADC values in the cancer lesions is consistent with this interpretation.

These findings are preliminary and must be interpreted within the limitations of the study design, including the small number of confirmed lesions, the absence of prostatectomy-based imaging-pathology correlation, and the patient-specific variability introduced by the internal blood reference assumption. They do not permit conclusions regarding diagnostic sensitivity, specificity, or clinical thresholds. Prospective validation in larger cohorts with whole-mount histopathological correlation, zone-specific analyses, and stratification by Gleason grades is required before the clinical utility of ^23^Na MRI-derived TSC can be established as a biomarker for prostate cancer characterization and risk stratification.

## Figures and Tables

**Table 1:** Characteristics of the patient collective stratified for PI-RADS categories, mean values and standard deviations. Gleason scores were not available for all patients with PI-RADS scores of 1–3 either since no biopsy was performed or due to loss of follow-up. Prostate volumes for calculation of PSAD were obtained from prostate segmentations.

PI-RADS	All	1–3	4	5
**Number of patients**	36	28	3	5
**Age**	66.5 ± 7.5	65.6 ± 7.1	62.7 ± 3.2	74.0 ± 7.5
**PSA**	9.1 ± 6.1	7.8 ± 3.9	6.7 ± 2.7	17.9 ± 10.2
**PSAD**	0.19 ± 0.16	0.15 ± 0.10	0.23 ± 0.06	0.43 ± 0.24
**Gleason (*n*)**	4 + 5: 3 4 + 3: 1 3 + 4: 5 3 + 3: 5	3 + 4: 3 3 + 3: 3	3 + 4: 1 3 + 3: 2	4 + 5: 3 4 + 3: 1 3 + 4: 1
**Gleason available (*n*)**	22	6	3	5

PSA—prostate specific antigen; PSAD—PSA density; *n*—number.

**Table 2:** Mean absolute tissue sodium concentration (TSC) of the whole prostate (WP), its peripheral zone (PZ), transition zone (TZ), suspicious lesions with PI-RADS 4 and 5 (lesions) and regions of interest in healthy prostate tissue contralateral to the suspicious lesions (contralateral). Values are given for all patients and stratified for patients without suspicious lesions (PI-RADS 1–3), suspicious lesions in the PZ and TZ (PI-RADS 4-5).

Patients	TSC [mM] WP	PZ	TZ	Lesions	Contralateral
**All patients**	39.0 ± 5.3	40.7 ± 6.0	37.5 ± 5.7	32.2 ± 5.5	36.1 ± 3.9
**No suspicious lesions** **(PI-RADS 1-3)**	39.8 ± 5.6	41.6 ± 6.0	38.3 ± 5.9	--	--
**Suspicious lesion in PZ** **(PI-RADS 4 and 5)**	35.0 ± 1.9	36.0 ± 4.1	33.1 ± 2.9	32.1 ± 5.8	36.3 ± 4.1
**Suspicious lesion in TZ** **(PI-RADS 4 and 5)**	40.5 ± 0.1	41.4 ± 0.4	39.0 ± 1.3	32.7 ± 4.2	36.1 ± 4.0

## Data Availability

The datasets presented in this article are not readily available due to privacy restrictions. Requests to access the datasets should be directed to fabian.tollens@medma.uni-heidelberg.de.

## Supplementary Materials

The following supporting information can be downloaded at: https://www.mdpi.com/article/10.3390/diagnostics16071064/s1, Table S1: Structured methodological comparison of published ^23^Na MRI studies in the human prostate.
