# Supplementary material for: Decreased Tissue Sodium Concentration in Suspected Prostate Cancer Detected by Internal-Reference 23Na MRI: A Prospective Exploratory Study
Source: Diagnostics (Basel). 2026 Apr 1;16(7):1064. doi: 10.3390/diagnostics16071064 (PMC13074163; doi:10.3390/diagnostics16071064)
Supplement: Supplementary file 1 [file diagnostics-16-01064-s001.zip › diagnostics-4209199-supplementary.pdf]

Supplementary Table S1. Structured methodological comparison of published ^{23}Na MRI studies in the human prostate.

Parameter	Barrett et al. 2018 Invest Radiol	Barrett et al. 2020 J Magn Reson Imaging	Broeke et al. 2019 J Magn Reson Imaging	Tan et al. 2024 Radiol Adv	Present study
**Acquisition & hardware**					
**Coil type**	Endorectal *Dual-tuned $^{1}H/^{23}Na$ ERC + clamshell TX coil*	Endorectal *^{23}Na ERC + ^{1}H body TX; ^{23}Na vs. ^{13}C ERC comparison*	External surface *Broadband surface coil + body TX*	External *Custom 2-loop butterfly coil (Ø 18 cm) + body TX*	External *16-ch birdcage TX + 16-ch flex array RX; dual-tuned $^{1}H/^{23}Na$*
**Field strength**	3 T	3 T	3 T	3 T	3 T
**Sequence / resolution**	3D GRE radial *Nominal resolution not reported*	3D GRE radial *Nominal resolution not reported*	3D GRE *~5 mm isotropic (nominal)*	3D radial UTE *~4–5 mm isotropic (nominal)*	3D radial density-adapted *5.1 × 5.1 × 5.1 mm³; acq. time 16 min*
**TSC quantification**					
**TSC reference method**	External phantom *NaCl vials integrated into ERC; spatial proximity to coil, not tissue*	External phantom *Phantom-based calibration; coil-integrated vials*	External phantom *External reference phantoms + B1 correction via phantom scan*	External phantom *Sensitivity map normalization; no fully absolute calibration reported*	Internal reference *Femoral blood vessels (assumed TSC = 81 mM); spatially proximate to prostate*
**Relaxation time correction**	Full *T1 + biexponential T2* for tissue and blood; IR sequence for intracellular Na separation*	Full *T1 + T2* corrections; IR for intracellular Na; bilateral coil comparison*	Partial/relative *Relative ΔTSC (%) vs. healthy tissue; no fully corrected absolute TSC reported*	Partial *Sensitivity normalization applied; full T1/T2* relaxation correction not reported*	Full *T1 + biexponential T2* for prostate and blood; B1⁻ correction via low-pass filter*
**TSC metric**	Absolute (mM)	Absolute (mM)	Relative (ΔTSC, %)	Absolute (mM)	Absolute (mM)
**Study population & reference standard**					
**n (analyzed)**	15 PCa patients	8 PCa patients	10 PCa patients	20 PCa + 6 controls	36 patients *8 with PI-RADS 4/5 and biopsy-confirmed PCa*
**Histological reference standard**	Prostatectomy *Whole-mount; imaging-pathology co-registration*	Prostatectomy *Whole-mount histology maps*	Prostatectomy *Gleason-graded whole-mount co-registration*	Biopsy *Targeted + systematic biopsy; no prostatectomy*	Biopsy only *Systematic TRUS-guided biopsy; PI-RADS 4/5 only; no whole-mount specimen*
**Patient selection**	Intermediate/high-risk PCa *Gleason 3+3 to 4+5; MRI-visible lesions; pre-prostatectomy*	Intermediate/high-risk PCa *Pre-prostatectomy; coil comparison design*	Biopsy-proven PCa *All Gleason grades; pre-prostatectomy*	Suspected or confirmed PCa *Mixed risk profile*	Clinically suspected PCa *PI-RADS 3–5; PSA ≥4 ng/mL; no prior treatment*
**Reported TSC values**					
**Healthy PZ (mM)**	39.2	39.2 / 40.1 *(^{23}Na / ^{13}C coil)*	— *(relative metric only)*	78.2 ± 14.1	40.7 ± 6.0
**Healthy TZ (mM)**	32.9	33.9 / 36.3 *(^{23}Na / ^{13}C coil)*	— *(relative metric only)*	80.9 ± 16.3	37.5 ± 5.7
**TSC in PCa lesions**	**↑ Elevated** *PZ tumor: ~45.0 mM; $p < 0.001$ vs. healthy PZ*	**↑ Elevated** *~45.4 mM (^{23}Na); ~49.4 mM (^{13}C); p = 0.02 / 0.002*	**↑ Elevated (relative)** *ΔTSC correlates with Gleason score (rs = 0.791, $p < 0.01$)*	**↓ Decreased** *TZ tumor: 64.0 ± 15.1 vs. 80.9 ± 16.3 mM; p = 0.0002 | PZ: not significant*	**↓ Decreased** *PZ lesions: 32.1 ± 5.8 mM vs. contralateral ROI; p = 0.018 | TZ lesions: below healthy TZ in both cases*
**Key methodological confounders**					
**Main confounders**	**ERC proximity bias:** phantom vials calibrate coil sensitivity, not tissue;	**ERC proximity bias** (as 2018 study); small n = 8;	**Relative metric** avoids absolute calibration errors; whole-mount RP gives	**External coil + partial correction:** higher absolute TSC vs. present study likely	**Internal reference:** reduces B1 inhomogeneity effects but introduces blood TSC variability

Parameter	Barrett et al. 2018 Invest Radiol	Barrett et al. 2020 J Magn Reson Imaging	Broeke et al. 2019 J Magn Reson Imaging	Tan et al. 2024 Radiol Adv	Present study
	inhomogeneous B1 field may overestimate peripheral TSC; whole-mount RP enables precise lesion mapping	coil comparison as primary aim	highest spatial precision; broadband surface coil limits deep tissue SNR	reflects sensitivity normalization differences; biopsy limits spatial precision	(haematocrit, serum Na); biopsy-only reference standard; largest prospective cohort in the field

Abbreviations: ERC = endorectal coil; TX = transmit; RX = receive; GRE = gradient echo; UTE = ultrashort echo time; IR = inversion recovery; ΔTSC = relative TSC change versus healthy tissue; RP = radical prostatectomy; TRUS = transrectal ultrasound; PCa = prostate cancer; PZ = peripheral zone; TZ = transition zone. Absolute TSC values are not directly comparable across studies due to differences in coil type, reference method, relaxation time correction, and B1 field homogeneity. The present study and Tan et al. 2024 are the only studies employing an external coil without an endorectal component; both report decreased TSC in confirmed PCa lesions.
